# Characterization and investigation of electrochemical and biological properties of antibacterial silver nanoparticle-deposited TiO_2_ nanotube array surfaces

**DOI:** 10.1038/s41598-023-31937-6

**Published:** 2023-03-22

**Authors:** Salih Durdu, Emine Yalçin, Atilgan Altinkök, Kültiğin Çavuşoğlu

**Affiliations:** 1 Industrial Engineering, Giresun University, Faculty of Engineering, 28200 Giresun, Turkey; 2 Department of Biology, Giresun University, Faculty of Science, 28200 Giresun, Turkey; 3 Turkish Naval Academy, National Defence University, 34940 Istanbul, Turkey

**Keywords:** Biomaterials, Biomaterials

## Abstract

The one of main reasons of the premature failure of Ti-based implants is infections. The metal- and metal oxide-based nanoparticles have very high potential on controlling of infections. In this work, the randomly distributed AgNPs-deposited onto well-ordered TiO_2_ nanotube surfaces were fabricated on titanium by anodic oxidation (AO) and electrochemical deposition (ED) processes. AgNPs-deposited nanotube surfaces, which is beneficial for bone tissue growth exhibited hydrophilic behaviors. Moreover, the AgNPs-deposited nanotube surfaces, which prevent the leaching of metallic Ti ions from the implant surface, indicated great corrosion resistance under SBF conditions. The electrochemical corrosion resistance of AgNPs-deposited nanotube surfaces was improved up to about 145% compared to bare Gr2 surface. The cell viability of AgNPs-deposited nanotube surfaces was improved. Importantly, the AgNPs-deposited nanotube surfaces exhibited antibacterial activity for Gram-positive and Gram-negative bacteria. Eventually, it can be concluded that the AgNPs-deposited nanotube surfaces possess high stability for long-term usage of implant applications.

## Introduction

The TiO_2_ nanotube arrays produced on titanium and its alloys have made a great impact in different areas such as solar cells, photocatalytic systems, water splitting, sensor, drug delivery and biomedical implants at last year’s^1–5^. The nanotubes that could be produced by anodic oxidation process refer to tubular surface structures in the nanometer scale. TiO_2_ nanotubes with nano-topography mimics bone surface morphology and contain bioactive and biocompatible oxide phases. TiO_2_ nanotubes have positive effect on cell adhesion, proliferation, differentiation and apatite formation^6^. Thus, they are regarded as being more biocompatible than bare titanium and its alloys^7–9^. This surface structure leads to increase surface area promoting greater osseo-integration. However, this is resulted in creating more favorable conditions for bacterial proliferation^10^.

Nano-structured surfaces which are expressed as “promising” to control bacterial adhesion and biofilm formation can be based on well- or random-formation^11^. Nano-structured surfaces usually provide low bacterial adhesion. Moreover, adhered bacteria have multiple contact sites with the surface. Adhered bacteria onto nanostructured surfaces suffer localized cell wall deformation. This adhesion onto contact sites can be resulted in causing bacterial death^12,13^.

AgNPs (silver nanoparticles) have many advantages such as good antibacterial activity, excellent biocompatibility, and satisfactory stability against to antibiotics and antibiotic organic antimicrobials for medical applications^14^. It is well known that AgNPs are one of the most commonly tested nanoparticles in nanobiotechnology due to their unique physical, chemical and biological properties^15^. AgNPs which affect cellular functions as well as structures exhibits very strong antibacterial effect compared to other Ag structures such as microparticles and ionic compounds^16^. Ag has a toxicity effect over extensive bacteria types. Thus, it has been widely exploited for its antibacterial applications^17^. The releasing of Ag^+^ ions is reduced by the penetration of AgNPs onto the nanotube structure. Thus, it was stated that AgNPs on the nanotube structure provides a long-lasting antibacterial capability and less cytotoxic Ag concentration compared to other Ag structures such as microparticles, ionic or compounds^18^.

There are many studies on the fabrication and investigation of Ag ions-, Ag-incorporated and AgNPs-doped TiO_2_ nanotubes for photocatalytic, sensor, agricultural and water splitting in the literature^19–24^. However, the investigations on biological properties of Ag ions-, Ag-incorporated and AgNPs-doped TiO_2_ nanotubes for medical applications are limited in the literature. To prevent biofilm formation and bacterial growth, AgNPs were doped onto TiO_2_ nanotube surfaces by various methods such as ultraviolet radiation, magnetron sputtering, immersion test, spray deposition, e-beam evaporation etc.^12,25–34^. Wei et al. evaluated Ag nanoparticles and Ag ions were both loaded on TiO_2_ nanotubes by immersion in AgNO3 solutions followed by ultraviolet light radiation^34^. Di et al. investigated the antibacterial and antioxidant properties of AgNPs incorporated tannic acid/nanoapatite composite coating on Ti by AO and immersion tests^35^. Zhao et al. evaluated antibacterial properties of AgNPs-doped nanotube coatings by AO and immersion processes^31^. Bilek et al. examined antibacterial activity of commercial AgNPs decorated–TiO_2_ nanotubes^36^. Hyuk Uhm et al. investigated AgNPs-doped nanotubes by AO and e-beam processes^33^. Sudhisha et al. evaluated antibacterial and biocompatibility of AgNPs doped poly(3,4-ethylene dioxythiophene) on nanotubes by AO and electro-polymerization^37^. Perumal et al. examined AgNPs incorporated polyaniline on TiO_2_ nanotube array by AO and electro-polymerization^6^. Staats et al. investigated antimicrobial potential and osteoblastic cell growth at selenium or silver incorporation onto nanotubes on Ti6Al4V^38^. However, the wettability, electrochemical, biocompatibility and antibacterial of AgNPs-deposited nanotube surfaces fabricated by AO and ED processes have not been investigated in detail whereas many investigations were carried out on Ag ions-, Ag-incorporated and AgNPs-doped nanotube surfaces.

This work aimed to develop and characterize a novel AgNPs-deposited nanotube surfaces on titanium substrates by combined AO and ED processes and to investigate biocompatiblitiy and antibacterial properties. All surfaces were characterized by XRD, SEM, EDX-mapping and contact angle goniometer. The electrochemical properties of the surfaces were investigated under SBF conditions by Tafel extrapolation methods. Furthermore, cell viability, alkaline phosphatase activity and blood protein adhesion were evaluated and antibacterial properties of the surfaces were assessed against to Gram-positive bacteria (*Streptococcus pyogenes, Bacillus subtilis, Staphylococcus aureus*) and Gram-negative (*Salmonella typhimurium, Pseudomonas aeruginosa, Escherichia coli*). Our results indicated that the AgNPs-deposited nanotube surfaces have an important role on electrochemical properties, biocompatiliblity and antibacterial properties. Thus, they should be addressed for designing antibacterial surfaces for implant applications.

## Experimental details

### Sample preparation and coating fabrication

Grade 2 titanium (ASTM F67 cp-Ti; Shaanxi Aone Titanium) plates (10 mm × 10 mm × 1 mm) were polished from 180# to 2000# sandpapers. Afterwards, all prepared plates were cleaned in an ultrasonic bath and dried.

The cp-Ti plates were anodized in 0.5% wt. NH_4_F and 5.0% vol. deionized water containing ethylene glycol-based solution at 50 V for 1 h below 30 °C by using a DC power supply (Good Will Instek PSU 400). They were ultrasonically cleaned in distilled water solution for 15 min and then dried in warm air (40 °C) for 1 min by heat gun. All AO coated samples were transferred to muffle furnace. After AO process, the heat treatment was carried out to transform from amorphous to crystalline form at 450 °C for 1 h as reported previous works^39,40^. The cp-Ti and Pt were served as positive and negative electrodes through AO process, respectively.

Antibacterial AgNPs on nanotube surfaces were deposited at a constant potential value of − 1 V for 0.5 min (Gr2-50 V-Ag-0.5 m), 1 min (Gr2-50 V-Ag-1 m) and 5 min (Gr2-50 V-Ag-5 m) by using a potentiostat/galvanostat device (Gamry 1010E). Nanotube-coated surfaces in an aqueous electrolyte consist of 10 mM AgNO_3_ (Merck, ACS, ISO, Reag. Ph Eur) were dipped and coated. The Ag structure was randomly deposited as nanoparticles on the nanotube surfaces by ED process. After ED process, all samples were cleaned in distilled water solution for 15 min by in an ultrasonic bath.

### Characterization of the surfaces

The surface morphology was taken at 20,000× and 50,000× of magnifications with using 15 kV of accelerating voltage by SEM (Hitachi SU 1510). The elemental distribution and elemental amount of the surfaces were investigated at 500× of magnification with using 15 kV of accelerating voltage by EDX-mapping and -area. The surface roughness (Sa) and topography were evaluated in the 2.5 µm × 2.5 µm with using a TAP-190 cantilever by a Nanosurf C3000 Atomic Force Microscopy (AFM). Average contact angles of the surfaces were investigated with a sessile constant drop technique by a Dataphysics OCA-15EC contact angle goniometer. All contact angles were measured within 1 min after the water droplets with 1 µL contacted the surfaces.

### Electrochemical test

Electrochemical tests were carried out in SBF solution^41^ at body temperature (36.5 °C) by using Tafel extrapolation test (Metrohm Autolab PGSTAT101 potentiostat/galvanostat). To determine Tafel potential values, the OCP (open circuit potentials) were initially measured for all samples. Tafel extrapolation test were carried out at 1 mV/s between − 500 and 500 mV of OCP. A conventional three-electrode cell such as the sample working, Ag/AgCl reference and Pt counter was used for electrochemical tests as described previous work^42^.

### Cell viability test

Biomaterials used in medical applications should be biocompatible with blood and should not exhibit cytotoxicity. The cytotoxic effect of each coating was determined by MTT test using RPMI 8866 (Cell Linehuman)-Lymphoblastoid cell cells. Cells were incubated at 37 °C (5% CO_2_) in a solution containing 2 mM Glutamine, 100 IU/mL penicillin, RPMI 1640, 10% Fetal Bovine Serum and 100 µg/mL streptomycin. Each coating (0.5 cm^2^) was sterilized in an autoclave and placed in cell culture wells. Media containing 2 × 10^5^ cells/mL cells was transferred to the wells. Coatings and cells were incubated for 48 h. Then, 200 µL of medium containing 20 µL of 5 µg/mL MTT and 200 µL of isopropanol containing 0.04 M HCl were added to each well. After 4 h of incubation, absorbances were measured at 570 nm by spectrophotometer. Cell viability was evaluated according to the positive control in which DMSO was used.

### Alkaline phosphatase activity

ALP activity is a significant indicator in determining the activity of osteoblastic cells. The principle of this test is based on the conversion of p-nitrophenyl phosphate to p-nitrophenol and phosphate by hydrolysis. SAOS-2 (primary osteogenic sarcoma, Cell Line human) cells were grown by incubating for 72 h in medium containing McCoy's 5A (l-Glutamine, serum-free), penicillin–streptomycin (1%) and fetal calf serum (15%). Coatings (0.5 cm^2^) and 2 × 10^5^ cells/mL of cells were interacted for 48 h. At the end of the interaction, cells were collected and washed with lysis buffer (250 µL, 0.5% v/v Triton X-100). Cells were incubated in 1 mM MgCl_2_ and Tris (pH 7.6), then centrifuged at 5000 rpm for 10 min. 50 µL of supernatant and 50 µL of ALP reagent were mixed and spectrophotometric measurements were performed at 410 nm.

### Artificial blood protein adhesion

Adhesion of blood proteins in blood compatibility of biomaterials is also one of the tested parameters. The adhesion of blood proteins to a hemocompatible surface reduces its compatibility. For this purpose, the interactions of the coatings with gamma-globulin, albumin and fibrinogen proteins were investigated. Commercially purchased albumin, gamma-globulin and fibrinogen proteins were prepared at 20 mg/mL concentration and incubated with the coating surfaces at 37 °C for 2 h in a batch system. After incubation, the coatings were washed twice with 15 mL of saline solution to remove surface proteins. Ultrasonic vibration at 35 kHz frequency for 5 min was applied to obtain the proteins that adhere to the coating surface. The concentrations of proteins, which separated from the surface and passed into the solution, were determined at 280 nm.

### Bacterial adhesion test

Bacterial adhesion assays were performed against Gram-positive bacteria (*Streptococcus pyogenes, Bacillus subtilis, Staphylococcus aureus*) and Gram-negative (*Salmonella typhimurium, Pseudomonas aeruginosa, Escherichia coli*). Bacterial suspension was prepared using 5 mL of sterile medium from bacterial stock cultures. 100 μL of the suspension adjusted according to 0.5 McFarland, was combined with broth medium and interacted with the coating surface (0.5 cm^2^). After incubation for 24 h at 37 °C, the coatings removed from the medium were washed twice with 15 mL of saline solution to remove bacteria that did not adhere to the surface. Each coating was taken into tubes containing 2 mL of physiological solution and subjected to ultrasonic vibration at 35 kHz frequency for 5 min. After sonication, the samples taken from the tubes were transferred to agar medium and colonies of each bacteria were counted after 24 h of incubation.

### Statistical analysis

Statistical analysis was performed using “IBM SPSS Statistics 22 SP” and all results are given as mean ± SD. p < 0.05 was considered statistically significant.

## Results and discussion

### The morphologies of the surfaces

**Figure 1:**
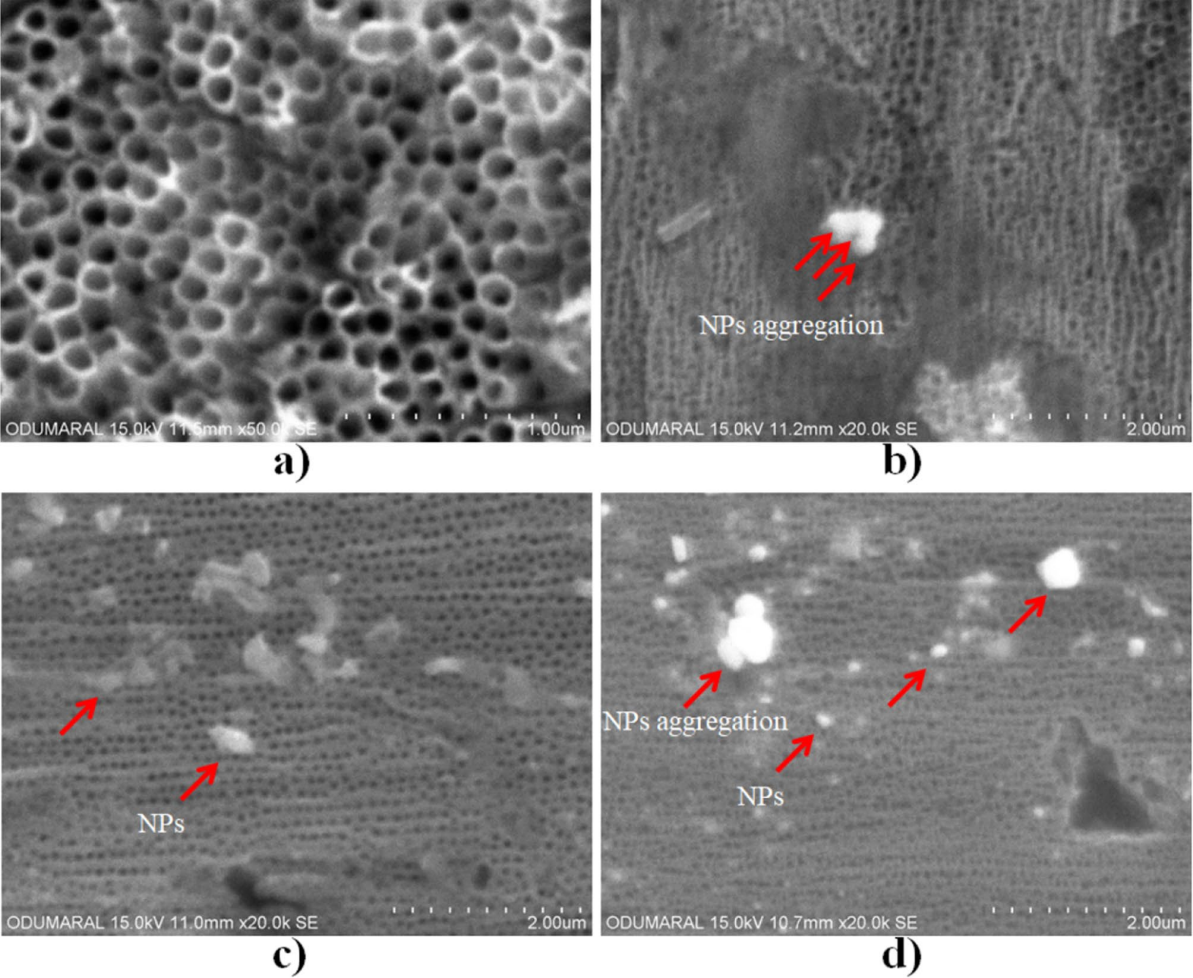
Surface morphologies of bare nanotube and AgNPs-deposited on nanotube surfaces at different times by ED process: (**a**) bare nanotube (50,000×), (**b**) 0.5 min, (**c**) 1 min and (**d**) 5 min.

The surface morphologies of bare nanotube array and AgNPs-deposited at 0.5 min, 1 min and 5 min on nanotube surfaces are given in Fig. 1. The average diameter was measured as approximately 110 nm for nanotubes on cp-Ti. A small addition of fluoride ions into ethylene glycol is one of the key factors to form well-ordered TiO_2_ nanotube structures. The typical amount of fluoride ion addition leading to well-ordered TiO_2_ array is between 0.3 and 0.5 wt% as reported in the literature^43^. F^−^ ions in the electrolyte, which have great reactivity, form many pits on the compact oxide layer through AO process. The chemical dissolution process is rapid in electrolytes containing F^-^ ions. This leads enlarging and deepening the pits and elongates them upward^44^. At the bottom of the pores, chemical dissolution continues to occur, thinning the oxide layer significantly^45^. As a result of these, TiO_2_ nanotube arrays form on titanium surface.

As seen in Fig. 1, the amount and size of brightly colored particles accumulated on the surface increase with the increasing time during the ED process. The sizes for 0.5, 1 and 5 min were measured as 85, 113 and 141 nm by Fiji software, respectively. At the first stages of the ED process, while the particles are finer and homogeneous, nanoparticles of different sizes are coated on the nanotube surfaces in a more non-uniform manner depending on the deposition rate with the increase in time. While some nanoparticles enclose a single or more tube structure, some nanoparticles agglomerate further and accumulate on the surface, closing several nanotube structures as shown in Fig. 1b–d. The formation mechanism of AgNPs contains the nucleation and growth of Ag structure during ED process. The top of nanotubes, which is potential nucleation sites for Ag nuclei, become nucleation and growth sites for AgNPs. Thus, Ag nanoparticles can be connected with each other by electrostatic interactions and transform into agglomerates with increasing ED processing time^46,47^. In fact, an Ag-based surface can be obtained by depositing more particles on the surface by covering the nanotube morphology completely by electro-deposition at much higher times. However, in order not to affect the nanotube morphology too much and not to exceed the cytotoxic limit, the nanoparticles were randomly deposited on the nanotube surfaces, especially at low times.

### Elemental analysis of the surfaces

**Figure 2:**
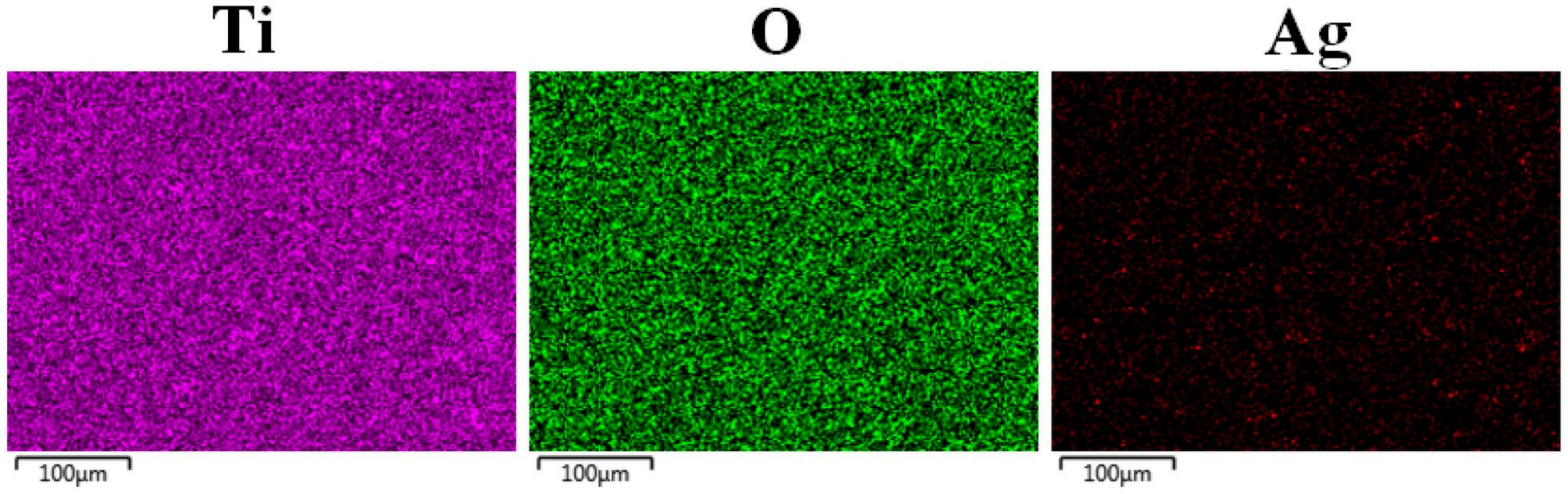
Elemental mapping images of AgNPs-deposited nanotube surfaces for 5 min.

**Figure 3:**
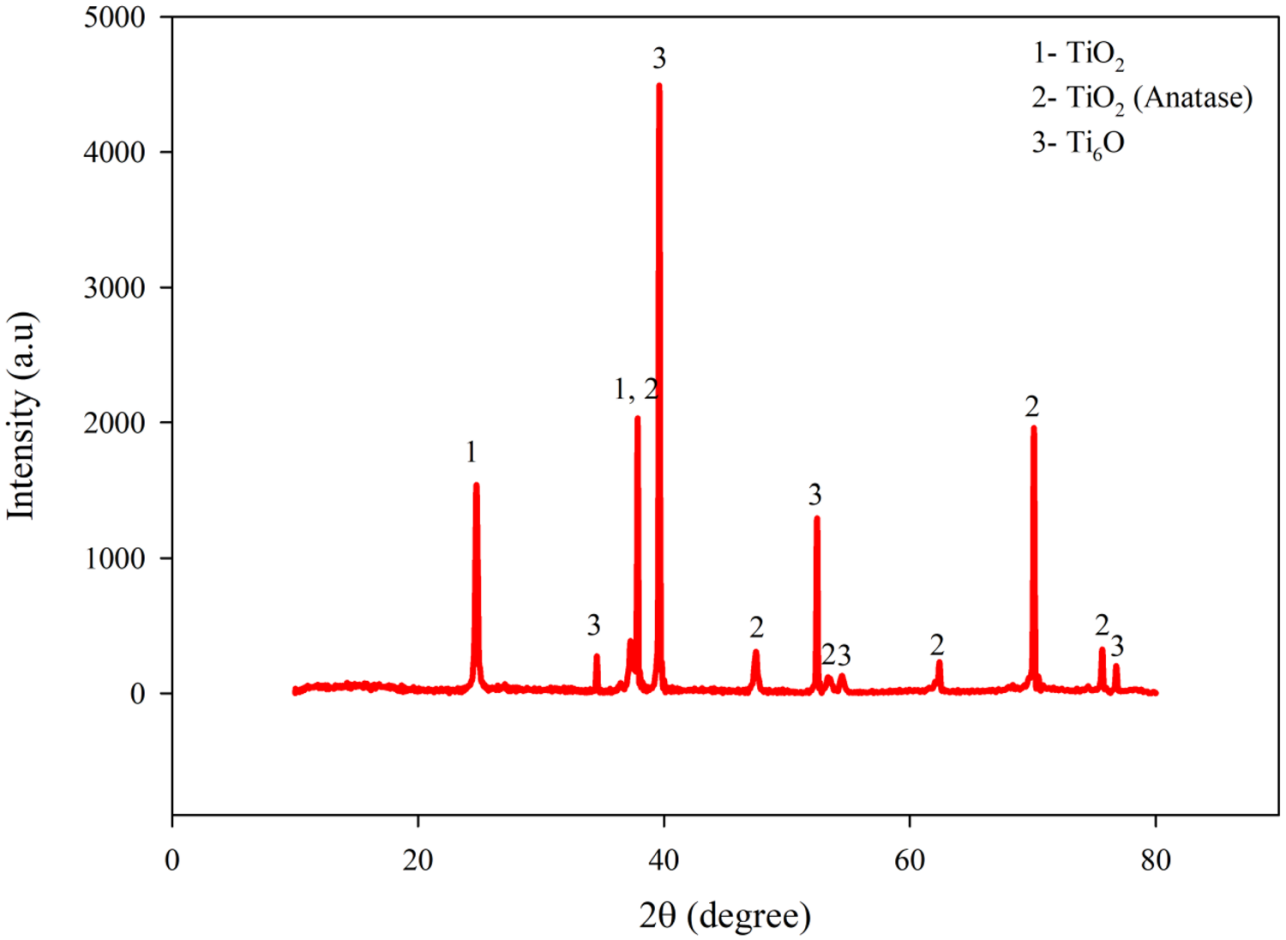
XRD spectra of AgNPs-deposited (Gr2-50 V-Ag-5 m) nanotube surfaces.

EDX-Mapping and EDX-area analysis were carried out to investigate the distributions and amounts of elements on the bare nanotube and AgNPs-deposited nanotube surfaces. EDX-Mapping analyzes of AgNPs-deposited nanotube surfaces produced on the cp-Ti surface are given in Fig. 2. In addition, the amounts of Ag elements observed on nanotube surfaces are given in the Table 1. As seen in Table 1, the amount of Ag structure increases since the more AgNPs deposits onto nanotube surface with increasing ED time. Due to the presence of the base TiO_2_ nanotube structure, Ti and O elements were detected in EDX analysis. However, in addition to TiO_2_ phase, the phase of Ti_6_O exists on the surface as seen in Fig. 3. Thus, Ti:O ratio is not 1:2 as shown in Table 1. Furthermore, the existence of Ag element was proved as observed as bright nanoparticles on SEM images. In the ED process, AgNPs accumulate randomly on the nanotube surfaces depending on the voltage and time. And, the deposition time values are kept as low as possible in order not to disturb the morphology of the surface and not to exceed the cytotoxic limit. The elemental values for the AgNPs structure were observed below the cytotoxic limit during ED process as reported in the literature^48,49^.

**Table 1:** Elemental amounts of bare nanotube surface and AgNPs-deposited nanotube surfaces (at. %).

Sample	Ti	O	Ag
Nanotube (50 V)	30.95	69.05	–
Gr2-50 V-Ag-0.5 m	32.03	67.88	0.08
Gr2-50 V-Ag-1 m	29.70	70.20	0.10
Gr2-50 V-Ag-5 m	29.35	70.47	0.18

### Phase structures of the surfaces

In order to observe possible Ag structure that may form on nanotube surface; AgNPs-deposited nanotubes produced at 5 min with the highest time was analyzed as seen in Fig. 3. The TiO_2_ (JCPDS card number: 96-152-6932), anatase-TiO_2_ (JCPDS card number: 96-720-6076) and Ti_6_O (JCPDS card number: 96-152-9956) phases were detected on AgNPs-deposited on nanotube surfaces. TiO_2_ nanotubes are usually exist amorphous form on Ti surfaces at room temperature. Amorphous TiO_2_ nanotubes are transformed into crystalline anatase or mixed crystalline anatase/rutile phases through annealing at various temperatures^50^. Amorphous/crystalline anatase phase transition begins to appear at 280 °C as reported in the literature^51^. Thus, at post-fabrication AO process, the heat treatment is applied on the nanotube surfaces for 60 min at 450 °C as described in experimental section. Thus, these oxide structures in the nanotube structures are transformed from amorphous to crystalline form without any morphological difference under low temperature conditions with by a diffusion mechanism. In our previous works^40,52^, the anodic oxidation mechanisms under the existence of fluoride ions were discussed in detail. The AO process is the electrolysis of water at the first reaction. Subsequently, a compact TiO_2_ layer is formed by dissolving of F-ions on bare Gr2 at the second reaction. Eventually, the pits which occur at the third reaction transform into nanotube structures at the suitable voltage and time through AO process. Although Ag structure was observed by EDX-mapping and EDX-area analysis, it could not be detected by XRD. This could be related that the amount of the deposited Ag structures on nanotube surfaces was kept at very low levels for XRD detection limits.

1 $${\text{2H}}_{{2}} {\text{O}} \leftrightarrow {\text{O}}_{{2}} + {\text{4H}}^{ + } + {\text{4e}}^{ - }$$

2 $${\text{Ti}} + {\text{O}}_{{2}} \leftrightarrow {\text{TiO}}_{{2}}$$

3 $${\text{TiO}}_{{2}} + {\text{6F}}^{ - } + {\text{4H}}^{ + } \leftrightarrow \left[ {{\text{TiF}}_{{6}} } \right]^{{{2} - }} + {\text{2H}}_{{2}} {\text{O}}$$

### Topography of the surfaces

Average surface roughness (Sa) values of the surfaces were given in Table 2. The average roughness values of AgNPs-deposited nanotube surfaces produced on nanotube surfaces vary between approximately 154.5–269.1 nm while bare Gr2 and bare nanotube surfaces are measured as 10.7 nm and 53.2 nm, respectively. The lowest roughness values were measured on bare Gr2 cp-Ti metal surfaces as expected. The roughness of nanotube surface produced at 50 V improves compared to bare Gr surface owing to the presence of nanotubular morphology. The surface roughness of the nanotube coating usually increases with voltage and time compared to bare substrates. Moreover, an extra contribution of AgNPs on nanotube surfaces increases the surface roughness.

**Table 2:** Average roughness (Sa) of the surfaces.

Potential (V)	Sa (nm)				
	Bare Gr2	Bare nanotube	0.5 min	1 min	5 min
50	10.7	53.2	269.1	154.5	167.1

### Surface wettability

**Figure 4:**
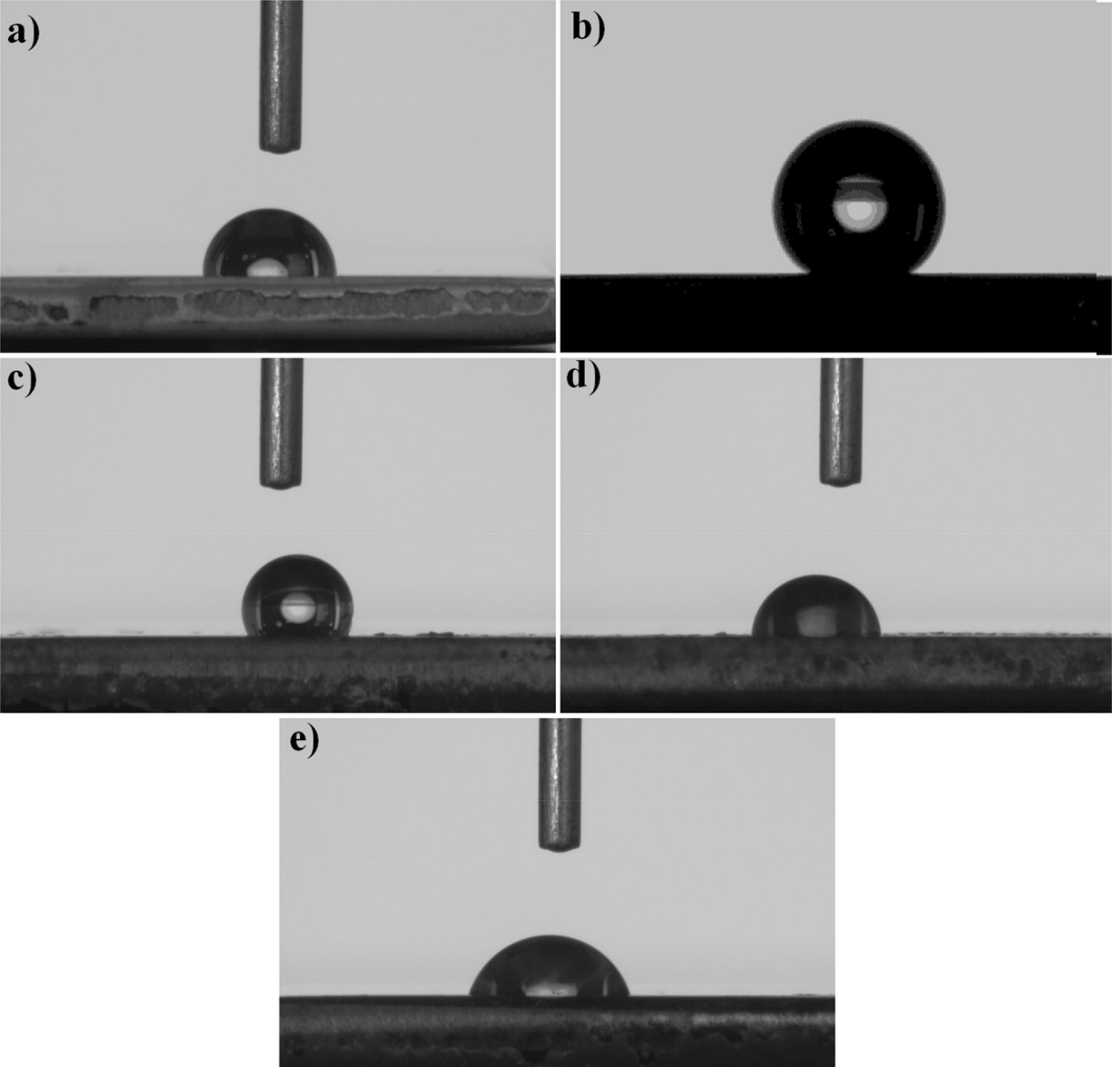
Contact angle images of the surfaces: (**a**) bare Gr2, (**b**) bare TiO_2_ nanotube, (**c**) Gr2-50 V-Ag-0.5 m, (**d**) Gr2-50 V-Ag-1 m and (**e**) Gr2-50 V-Ag-5 m.

The hydrophilic/hydrophobic properties of bare Gr2, bare nanotube and AgNPs-deposited nanotube surfaces by the contact angle goniometer (see in Fig. 4). The surfaces with low contact angles show hydrophilic character while the surfaces with high contact angle refer to hydrophobic behavior. The hydrophilic or hydrophobic properties of the surface vary from many parameters such as the surface energy, roughness, porosity and surface charges. The average contact angles of bare Gr2 and bare nanotube surfaces were measured 79.2° ± 0.2 and 140.2° ± 0.2, respectively. Thus, the bare Gr2 surface indicates low wetting angle value respect to the nanotube surface. The nanotube surfaces usually indicate hydrophilic/super hydrophilic behaviors as reported in the literature. The possible reason is anatase causes hydrophilic properties to be generated or another possibility can be the enlarged gap between nanotubes offering more space for liquid penetration^53^. This refers to the classical Wenzel model^54^. Wenzel model assumes that a water droplet fills up a rough surface. And, a fully wetted occurs, which depends on the roughness factor and the surface free energy. In other words, the Wenzel model suggests a classical situation of a water droplet on a ‘flat’ surface by roughness parameters. Thus, a hydrophilic/super-hydrophilic surface is created as a material with high surface energy combines with micro- and nanoscale roughness. However, just like in this work, Yang et al. observed the increase of contact angles from 77° to 141° under atmospheric air conditions at post-annealing of TiO_2_ nanotubes^55^. This wetting behavior can be ascribed to the replacement of the chemisorbed OH^-^ groups with O_2_ gaseous^56^ and the adsorption of organic contaminants on the TiO_2_ nanotube layer in atmospheric air conditions^55^. Thus, contact angles of TiO_2_ nanotubes are greater than one of bare Gr2 surface and previous works^55,57,58^. This behavior can be attributed to the well-ordered nanotube structure and the greater surface area. This result also refers to the Cassie-Baxter situation. The Cassie-Baxter situation states that the liquid forms a contact line with the air trapped below the contact line on the rough surface. Moreover, TiO_2_ surfaces are often partially hydroxylated and therefore have a polar structure. This resulted in the measurement of relatively low contact angles^59^. However, there is an exception for TiO_2_ nanotube arrays as given in results. The droplet is initially contacted on the protective passive TiO_2_ oxide layer on bare Gr2 surface. This layer exists naturally on bare Gr2 surface and the surface exhibits hydrophilic character due to its partially hydroxylated polar structure. However, after the droplet contacts on bare TiO_2_ nanotube surface, the atmospheric gases inside the tubes create resistance for a short time and prevent wettability of the surface. Eventually, this results in higher contact angles. Thus, TiO_2_ nanotube arrays indicate hydrophobic behavior compared to bare Gr2 surfaces.

In addition, the average contact angle values of AgNPs-deposited nanotube surfaces for 0.5 min, 1 min and 5 min were obtained as 118.5° ± 0.1, 86.6° ± 0.6 and 73.2° ± 0.5, respectively. The wettability (hydrophilic properties) of AgNPs-deposited nanotube surfaces is improved by increasing ED deposition time. However, except for Gr2-50 V-Ag-5 m, the contact angle values for AgNPs-deposited TiO_2_ nanotubes produced at low ED treatment times are higher than for bare Ti surface. The hydrophilic/hydrophobic surface affects cell adhesion and spreading. While some studies in the literature indicate that cell proliferation is easy on hydrophilic surfaces, some studies have shown that cell adhesion is better on hydrophobic surfaces^60,61^. However, the number and amounts of AgNPs on TiO_2_ nanotube layer increase with increasing ED deposition time as seen in Fig. 1. Thus, the number of defective sites increases with increasing AgNPs content for 5 min and this led to the improvement of hydrophilicity^62^. It could be stated that the deposition of AgNPs on TiO_2_ nanotube layer improves hydrophilic properties and wettability.

### Electrochemical corrosion resistance of the surfaces

**Figure 5:**
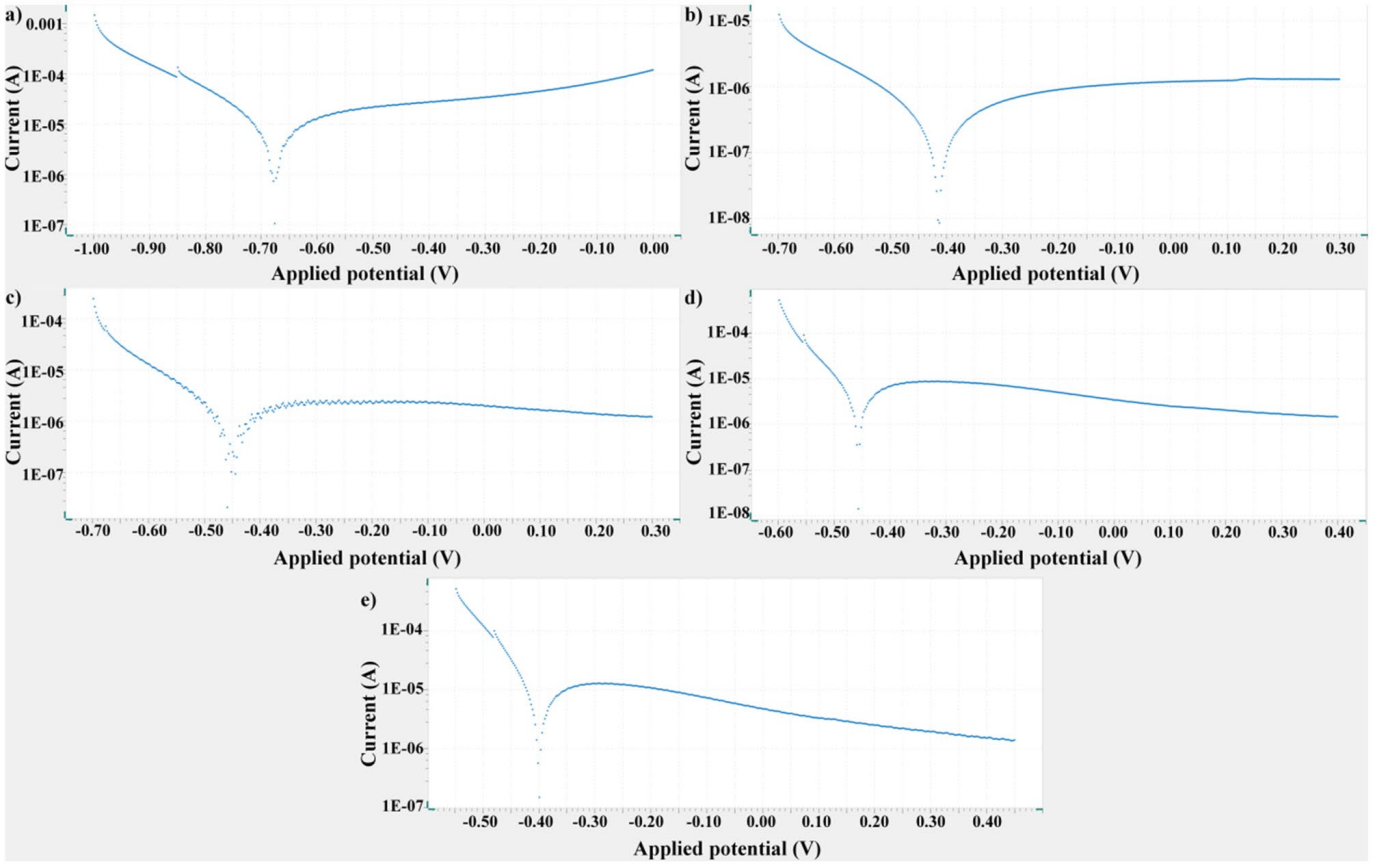
Tafel polarization curves of bare Gr2, bare nanotube and AgNPs-deposited nanotube surfaces.

The OCP and Tafel extrapolation test results (such as polarization resistance (Rp), corrosion potential (E_cor_) and corrosion current density (i_cor_)) of and bare Gr2, bare nanotube and AgNPs-deposited nanotube surfaces are given in Table 3. Also, Tafel extrapolation polarization curves were shown in Fig. 5. It is well known that i_cor_ values are inversely proportional to the corrosion rate^63^. Bare Gr2 surfaces show a higher corrosion current density value and lower corrosion resistance by passivizing at lower potential values compared to bare nanotube and AgNPs-deposited nanotube surfaces. In general, the corrosion resistance depends on the characteristic properties of the surfaces such as the phase structure, elemental amount, surface morphology and wettability.

**Table 3:** Corrosion parameters obtained from polarization curves taken at 1 mV/s scanning speed of bare Gr2, bare nanotube and AgNPs-deposited nanotubes at 36.5 °C in 1.0X SBF.

Samples	OCP (V)	E_kor_ (V)	i_kor_ (A/cm^2^)	Rp (ohm)
Bare-Gr2	− 0.460	− 0.672	2.57 × 10^–6^	6160.4
Bare-Gr2-50 V	− 0.214	− 0.413	1.82 × 10^–7^	14,284.0
Gr2-50 V-Ag-0.5 m	− 0.180	− 0.458	3.77 × 10^–7^	40,755
Gr2-50 V-Ag-1 m	− 0.107	− 0.456	1.35 × 10^–6^	7402.5
Gr2-50 V-Ag-5 m	− 0.065	− 0.400	2.07 × 10^–6^	3886.4

The passive oxide layer naturally formed on the bare Gr2 surface causes passivation behavior. TiO_2_ nanotube layer is a ceramic-based structure. The low electrical conductivity of TiO_2_ nanotube layer reduces charge transport and improves the electrochemical barrier properties compared to bare Gr2. The existence of oxide-based shielding coating can decrease the accessibility of the SBF solution to the bare Gr2 surface^64^. In addition, surface wettability is an important factor for corrosion resistance. A hydrophobic surface can effectively prevent the corrosive medium from permeating into the interface between the passivation coatings and the substrate. Thus, hydrophobicity inhibits corrosion process^65^. Predominantly; protective oxide nanotube layer in the AgNPs-deposited nanotube structures creates a barrier between the substrate metal and the atmosphere. This leads to reduce ion release and to increase corrosion resistance. Therefore, AgNPs-deposited nanotube surfaces show lower corrosion current density than the bare Gr2 metal. However, with the increase of the deposition time for the AgNPs structure, the corrosion current density gradually increased. AgNPs can be dissolved under SBF conditions and ionized to the SBF. The dissolution of AgNPs on nanotubes may be increased under SBF conditions with increasing ED time. And, this resulted in decreasing the corrosion resistance with increasing of deposition time whereas AgNPs-deposited nanotube surfaces indicated great corrosion resistance compared to bare Gr2 surface. This may be originated in ionization and dissolution of Ag structure under SBF with increasing ED time. The decrease in corrosion resistance could be due to rapid dissolution along under SBF conditions with agglomeration of Ag structure deposited on the nanotube surface with increasing deposition time. Thus, corrosion resistance of Gr2-50 V-Ag-0.5 min is greater than one of 1 min and 5 min.

### Cell viability (cytotoxicity) of the surfaces

**Figure 6:**
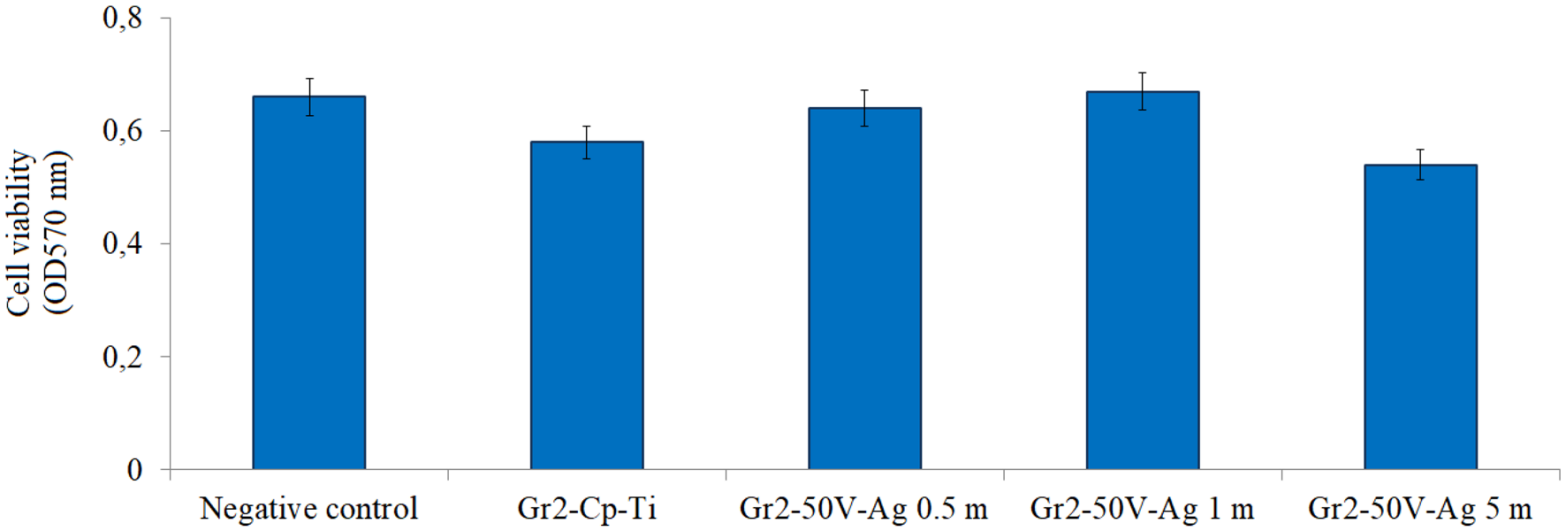
MTT test results of bare Gr2 and AgNPs-deposited nanotube surfaces. Different letters shown in the graph indicate statistical significance (p < 0.05).

The osseointegration of an implant is closely related with the interfacial response of cells contacted to the implant surface. The longevity of an implant preliminary is decided by the interaction and response between cells and biomaterial surface. Inadequately adherent cells inhibit tissue repair and regeneration, resulting in implant failure^6,66^. The changes in the viability of lymphoblastoid cells that adhere to AgNPs-deposited nanotube surfaces are given in Fig. 6. All AgNPs-deposited nanotube surfaces showed a decrease in cell viability compared to the negative control. Compared to bare Gr2 surface, cell viability varies depending on the amount of AgNPs on nanotube surface. Among AgNPs nanotube surfaces, the cell viability is the most for Gr2-50 V-Ag-1 m. Cell viability determined on Gr2-50 V-Ag-0.5 m and Gr2-50 V-Ag-1 m surfaces was found to be at the same level as negative control values. This shows that the modifications applied to Gr2-50 V-Ag-0.5 m and Gr2-50 V-Ag-1 m surfaces increase cell viability compared to bare Gr2 surfaces. Briefly, except for Gr2-50 V-Ag-5 m, the cell viability of AgNPs-deposited nanotube surfaces was higher than bare Gr2 surface and they indicate non-toxic character. Unlike other Ag coated surfaces, cell viability decreased on Gr2-50 V-Ag-5 m surfaces. While the Gr2-50 V-Ag-5 m surface did not cause any change in cell viability compared to the bare Gr2 surface (p > 0.05), it caused a decrease compared to the negative control (p < 0.05). This indicates that low levels of AgNPs-deposited nanotube surfaces stimulate cell viability and cause a decrease in viability as Ag density increases on the surface. Similarly, Fiedler et al. reported that the proliferation of osteoblastic cells increased on the surface of titanium alloys coated with Ag ion implantation and high-level Ag implantation on the surface reduced the cell proliferation^67^.

### Alkaline phosphatase activity of the surfaces

**Figure 7:**
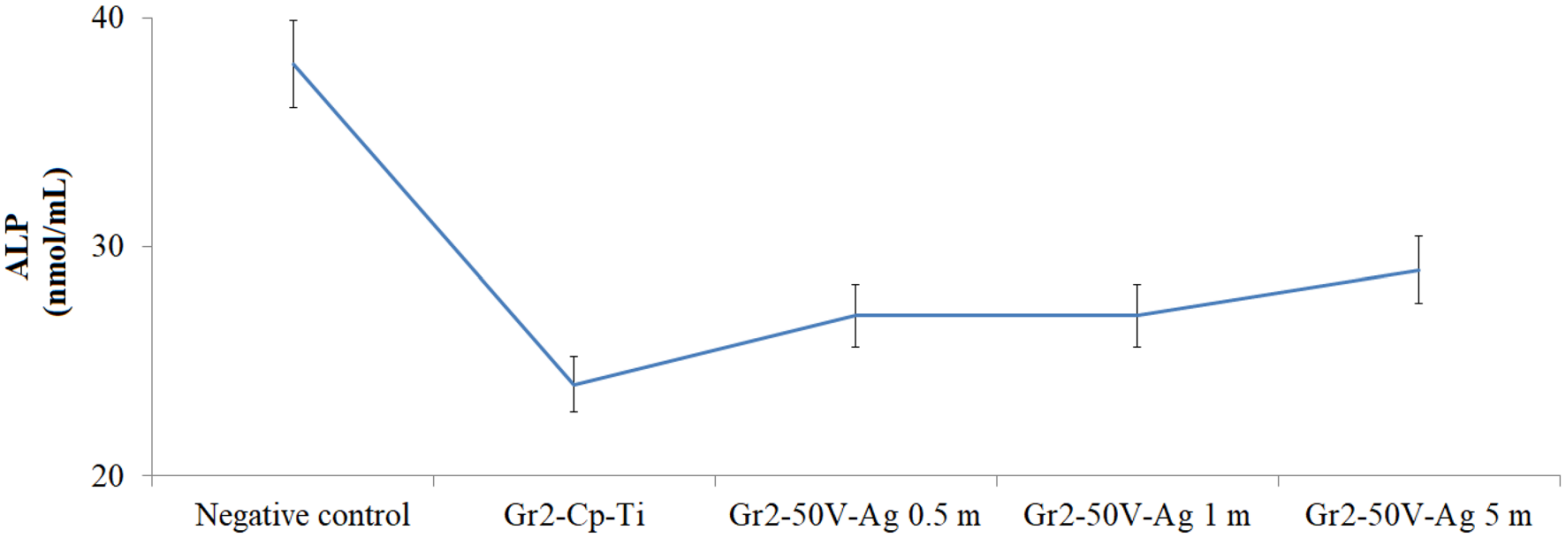
ALP activity in cells adhering to AgNPs-deposited nanotube surfaces.

ALP activities of cells adhering to bare Gr2 and AgNPs-deposited nanotube surfaces are given in Fig. 7. The ALP activity on all AgNPs-deposited nanotube surfaces was decreased compared to the negative control. However, an increase in ALP level on all AgNPs-deposited nanotube surfaces was observed compared to bare Gr2 surfaces. The highest ALP activity was obtained with Gr2-50 V-Ag-5 m, and similar levels of ALP activity were detected on Gr2-50 V-Ag-0.5 m and Gr2-50 V-Ag-1 m coatings.

When ALP activity and cell viability are evaluated together, cell viabilities and ALP levels increased on Gr2-50 V-Ag-0.5 m and Gr2-50 V-Ag-1 m surfaces. But on the Gr2-50 V-Ag-5 m surface, cell viability decreased, while ALP activity increased. Although ALP indicates the presence of osteoblast cells and new bone formation, it is present in many cells. ALP activity in a cell is related to cell viability and may vary with the conditions to which the cell is exposed. In other words, the cell can increase enzyme activity as an adaptation/tolerance mechanism against external factors that reduce its viability. For example, stopping the cell cycle in the G_0_/G_1_ phase causes an increase in ALP activity. In other words, cells can increase ALP activity against inhibition of cell proliferation. It is well known that Ag is more toxic compared to other antibacterial elements such as Cu and Zn. The surface of AgNPs attached to nanotubes is cytotoxic at an increased concentration of Ag due to the releasing of high content of Ag^+^ ions^68^. However, Ag has more advantage over a broad spectrum of bacteria respect to other antibacterial agents such as Cu and Zn. Sedelnikova et al. reported that cytotoxic effect occurred and ALP activity decreased in fibroblast cells adhering to Zn-, Cu- and Ag-stored titanium surfaces^69^.

### Blood proteins adsorption of the surfaces

The results of protein adsorption on bare Gr2 and AgNPs-deposited nanotube surfaces are given in Table 4. For AgNPs-deposited nanotube surfaces, the highest protein adsorption was obtained at fibrinogen while the lowest adsorption was observed at globulin. The protein adsorption on AgNPs-deposited nanotube surfaces was lower than on bare Gr2 surfaces. In other words, deposition of Ag caused a reduction in the adhesion of blood proteins. The lowest protein adhesion was detected in Gr2-50 V-Ag-5 m. Albumin, globulin and fibrinogen adhesion decreased 36.1%, 34.7% and 25%, respectively, in Gr2-50 V-Ag-5 m compared to bare Gr2 surfaces. Surfaces that are resistant to blood protein adsorption are quite successful in terms of hemocompatibility. Protein adsorption to the surface can be controlled by modifications in the surface properties. Therefore, it is crucial to control an implant's surface chemistry, especially its composition, in order to produce a specific surface with a well-defined biological reaction^70^. Undesirable reactions (such as coagulation) will occur after the adhesion of blood proteins when the surface comes into contact with blood. Therefore, decreased adhesion of blood proteins increases blood compatibility. Adhesion of extracellular proteins involved in cell–cell adhesion is tested for cells to settle on the surface and proliferate on the surface.

**Table 4:** Protein adsorption on bare Gr2 and AgNPs-deposited nanotube surfaces.

Sample	Albumin (mg/cm^2^)	Fibrinogen (mg/cm^2^)	Gamma-globulin (mg/cm^2^)
Gr2	19.21 ± 0.53^a^	17.72 ± 0.85^a^	14.91 ± 0.29^a^
Gr2-50 V-Ag-0.5 m	17.13 ± 0.50^b^	17.65 ± 0.70^a^	12.99 ± 0.81^b^
Gr2-50 V-Ag-1 m	14.91 ± 0.43^c^	15.83 ± 0.53^b^	10.81 ± 0.75^c^
Gr2-50 V-Ag-5 m	12.27 ± 0.19^ cd^	13.29 ± 0.49^bc^	9.73 ± 0.44^ cd^

Values are shown as mean ± SD. Means shown with different letters in the same column are statistically significant (p < 0.05).

### Antimicrobial activity of the surfaces

**Figure 8:**
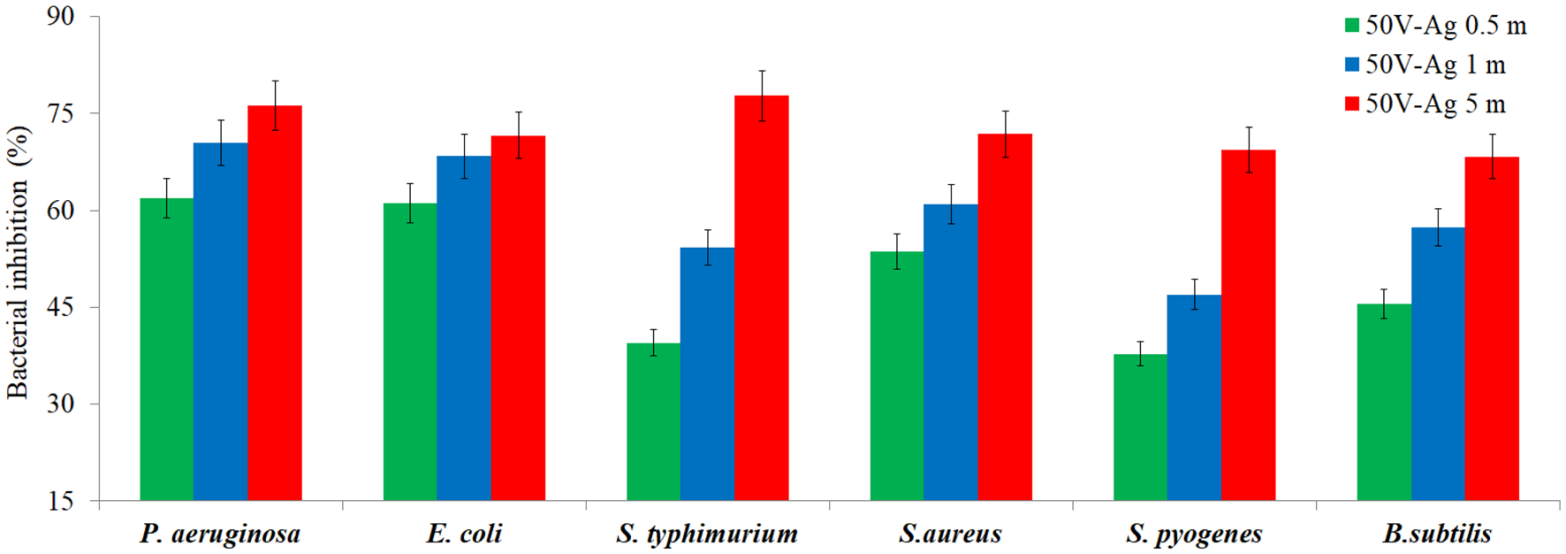
Percentage of bacterial inhibition of AgNPs-deposited nanotube surfaces.

**Figure 9:**
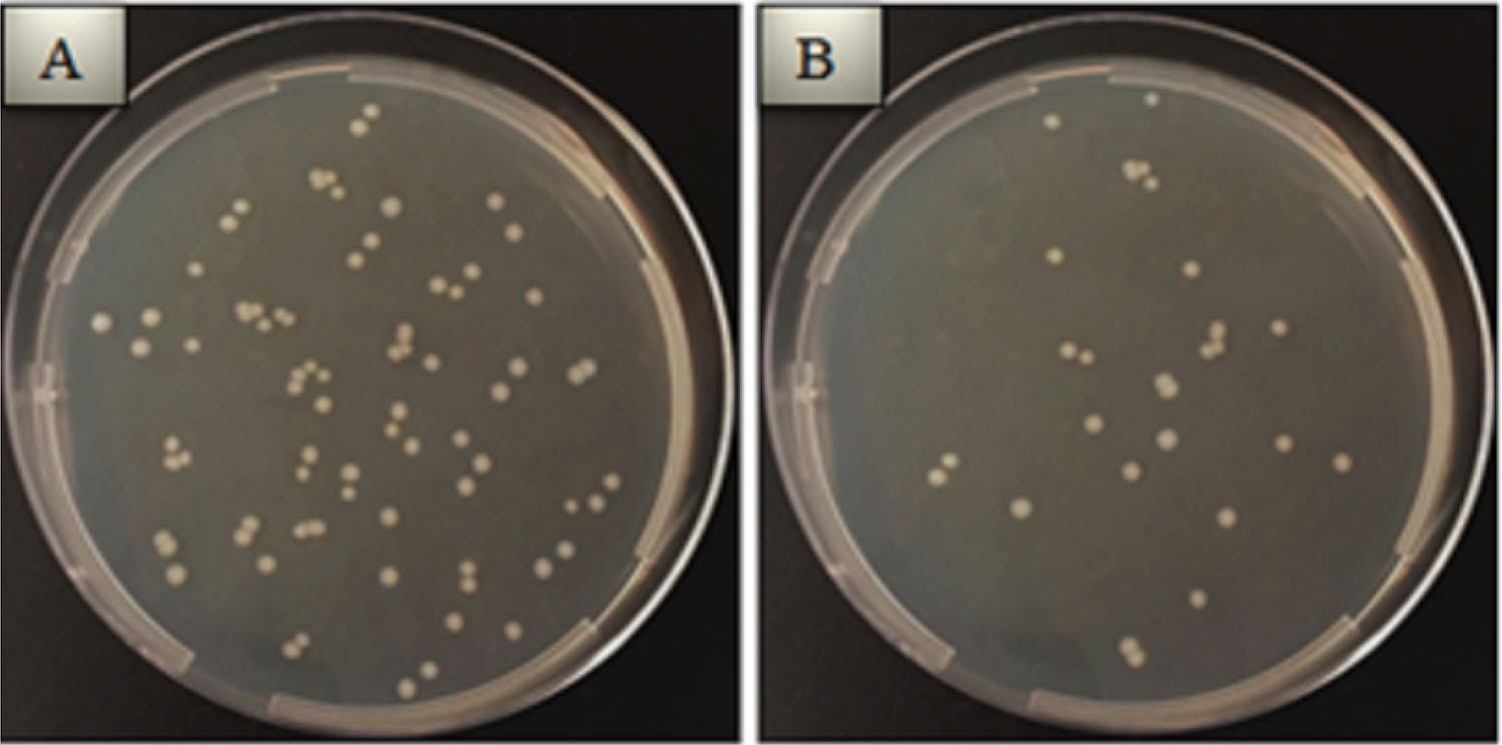
Reduction in bacterial colonies after re-culturation in samples with the highest antibacterial activity on AgNPs-deposited surfaces (**A**) *S. typhimurium* viability after re-culture on bare Gr2 surfaces, (**B**) *S. typhimurium* viability after re-culture on Gr2-50 V-Ag-5 m surfaces.

Inhibition percentages of AgNPs-deposited nanotube surfaces for different gram-positive and gram-negative bacteria are given in Fig. 8. The Ag deposition on nanotube surfaces lead to decrease in the level of bacteria colonies. For all bacteria types, the number of active colonies decreases with increasing AgNPs deposition time. For all AgNPs-deposited nanotube surfaces, the highest antibacterial activity was observed against Gram negative bacteria. Bacterial inhibition was achieved in the range of 68.3–71.7% in Gram-positive bacteria and in the range of 71.6–78.1% in Gram-negative bacteria. The highest bacterial inhibition was obtained on surface of 50 V-Ag-5 m surface against *S. typhimurium* as seen in Figs. 8 and 9.

The antibacterial mechanism of Ag structure which leads to bacterial death has not been completely clear. Antibacterial activity is not based solely on the release of Ag^+^ ions from AgNPs^71^. Although the antibacterial mechanism of AgNPs is still not fully elucidated, several studies have been conducted involving the antibacterial effect of modified TiO_2_ on Gram-positive/Gram-negative bacteria species^72^. Initially, AgNPs bind to the bacterial cell wall and leak into it. This leads to cause physical changes and damage the cell wall, resulting in leakage of cell contents. Subsequently, the bacteria are destructed by AgNPs^73,74^. The antibacterial effects of AgNPs-deposited nanotube surfaces can be explained by the toxic effect of Ag on bacterial cells. The Ag interacts with thiol groups of bacterial macromolecules, inhibiting enzymes and stopping respiratory reactions^75^. Electrostatic interactions between the positively charged Ag and the negatively charged membrane in contact of Ag-coated surfaces with bacteria cause physical damage to the bacterial membrane and further cidal effects. The denaturation of protein molecules, loss of function and inhibition of DNA synthesis occur when Ag enters into the bacterial cell^76,77^. As a cumulative result of all these effects on the bacterial cell, the cell cycle is disrupted and proliferation is reduced. Eventually, a decrease in colony formation occurs. It is well known that the Ag has a bacteriostatic/cidal effect by inhibiting phosphate uptake in *E. coli* and stopping the release of molecules such as phosphate, mannitol, succinate, proline and glutamate. In *S. aureus*, it has been reported that Ag prevents bacterial growth by damaging the bonds, glycan chains and amino acids in the peptidoglycan structure^78,79^. One of the important results obtained in this study is that AgNPs-deposited nanotube surfaces show stronger antibacterial effect against Gram negative bacteria. This selectivity in antibacterial activity can be explained by the differences in cell structure of Gram-positive and Gram-negative bacteria. This result is also supported by similar studies in the literature. Durdu et al. reported that there was a 67.2% decrease in the viability of *E. coli* and a 57.8% decrease in the viability of *S. aureus* and, Ag was more effective in Gram-negative bacteria^80^. Feng et al. reported that *E. coli* is more sensitive to Ag toxicity than *S. aureus*. This result is related to the defense system and cell structure of *S. aureus*^77^.

## Conclusion

The randomly distributed AgNPs-deposited TiO_2_ nanotube surfaces were fabricated on titanium by AO and ED processes. The wettability test confirms that AgNPs-deposited nanotube surfaces are hydrophilic compared to bare Gr2 surface. Moreover, the AgNPs-deposited nanotube surfaces, which prevent the leaching of metallic Ti ions from the implant surface, indicated greater corrosion resistance compared to bare Gr2 surface under SBF conditions. Except for Gr2-50 V-Ag-5 m, the cell viability of AgNPs-deposited nanotube surfaces was improved to bare Gr2 surface and it can be stated that they are non-toxic. Importantly, the AgNPs-deposited nanotube surfaces exhibited antibacterial activity for Gram-positive and Gram-negative bacteria. Eventually, it can be concluded that the AgNPs-deposited nanotube surfaces possess high stability for long-term usage of implant applications.

## Data Availability

The datasets used and/or analyzed during the current study are available from the corresponding author on reasonable request.
